# Birth outcomes in mothers with hypertensive disorders and polycystic ovary syndrome: a population-based cohort study

**DOI:** 10.1093/hropen/hoad048

**Published:** 2023-12-04

**Authors:** Xinxia Chen, Mika Gissler, Catharina Lavebratt

**Affiliations:** School of Nursing and Rehabilitation, Cheeloo College of Medicine, Shandong University, Jinan, Shandong, China; Department of Molecular Medicine and Surgery, Karolinska Institutet, Stockholm, Sweden; Center for Molecular Medicine, Karolinska University Hospital, Stockholm, Sweden; Department of Molecular Medicine and Surgery, Karolinska Institutet, Stockholm, Sweden; Center for Molecular Medicine, Karolinska University Hospital, Stockholm, Sweden; Department of Knowledge Brokers, Finnish Institute for Health and Welfare, Helsinki, Finland; Department of Molecular Medicine and Surgery, Karolinska Institutet, Stockholm, Sweden; Center for Molecular Medicine, Karolinska University Hospital, Stockholm, Sweden

**Keywords:** polycystic ovary syndrome, hypertensive disorder, pre-eclampsia, preterm birth, small for gestational age, large for gestational age

## Abstract

**STUDY QUESTION:**

Is polycystic ovary syndrome (PCOS) associated with higher risks of extreme birth size and/or preterm birth in mothers with different hypertension types?

**SUMMARY ANSWER:**

PCOS was associated with additional risks of preterm birth in mothers with chronic hypertension and in singleton pregnancies of mothers with pre-eclampsia, and with higher risks of offspring born large for gestational age (LGA) in mothers with gestational hypertension.

**WHAT IS KNOWN ALREADY:**

Women with PCOS are more likely to develop gestational hypertension, pre-eclampsia, and chronic hypertension. Although adverse birth outcomes have been frequently reported in mothers with PCOS, such associations in the setting of a hypertensive disorder remain unknown.

**STUDY DESIGN, SIZE, DURATION:**

This is a population-based cohort study including all live births 2004–2014 in Finland (n = 652 732). To ensure diagnosis specificity, mothers with diagnoses that could cause signs and symptoms resembling PCOS were excluded.

**PARTICIPANTS/MATERIALS, SETTING, METHODS:**

Maternal diagnoses of PCOS, gestational hypertension, chronic hypertension, and pre-eclampsia were identified from the Finnish national registries. Generalized estimating equation and multivariable logistic regression were used to assess the adjusted odds ratio (aOR) and 95% CIs of preterm birth, very preterm birth, and offspring being small for gestational age (SGA) or LGA in hypertensive mothers with or without PCOS, using normotensive mothers without PCOS as reference.

**MAIN RESULTS AND THE ROLE OF CHANCE:**

Of 43 902 (6.7%) mothers with hypertensive disorders, 1709 (3.9%) had PCOS. Significant interactions were detected for PCOS with hypertension on preterm birth, very preterm birth, offspring born SGA and LGA (*F*_preterm_ =* *504.1, *P*_interaction_ < 0.001; *F*_very preterm_ =* *124.2, *P*_interaction_ < 0.001; *F*_SGA_ =* *99.5, *P*_interaction_ < 0.001; *F*_LGA_ =* *2.7, *P*_interaction_ = 0.012, respectively). Using mothers with no hypertensive disorder and no PCOS as reference, the risks of preterm and very preterm birth were overrepresented in non-PCOS mothers with chronic hypertension or pre-eclampsia. PCOS was associated with higher risks of preterm birth (aOR_PCOS_ 4.02, 3.14–5.15 vs aOR_non-PCOS_ 2.51, 2.32–2.71) in mothers with chronic hypertension, with significant interaction between the exposures (*F *=* *32.7, *P*_interaction_ < 0.001). PCOS was also associated with a higher risk of preterm birth in singleton pregnancies of mothers with pre-eclampsia (aOR_PCOS_ 7.33, 5.92–9.06 vs aOR_non-PCOS_ 5.72, 5.43–6.03; *F *=* *50.0, *P*_interaction_ < 0.001). Furthermore, the associations of PCOS comorbid with chronic hypertension or pre-eclampsia was detected also for spontaneous births. Moreover, the risk of offspring LGA was higher in mothers with PCOS and gestational hypertension although lower in those with gestational hypertension alone (aOR_PCOS_ 2.04, 1.48–2.80 vs aOR_non-PCOS_ 0.80, 0.72–0.89; *F *=* *9.7, *P*_interaction_ = 0.002), whereas for offspring SGA, the risks were comparable between hypertensive mothers with and those without PCOS.

**LIMITATIONS, REASONS FOR CAUTION:**

Information on medication treatment, gestational weeks of onset for pre-eclampsia and gestational hypertension, weight gain during pregnancy, and PCOS phenotypes were not available. All diagnoses were retrieved from registries, representing only those seeking medical care for their symptoms. The ICD-9 codes used to identify PCOS before year 1996 are known to underestimate the prevalence of PCOS, while the inclusion of anovulatory infertility as PCOS might introduce an overrepresentation bias, although PCOS constitutes 80% of anovulatory infertility. The risk of very preterm birth in relation to maternal PCOS and hypertensive disorders should be interpreted with caution owing to limited sample sizes. Multifetal pregnancies among maternal PCOS were too few for a subgroup analysis. Moreover, ART included IVF/ICSI only. Potential effects of other treatments, such as ovulation induction, were not examined.

**WIDER IMPLICATIONS OF THE FINDINGS:**

PCOS was associated with additional risks of preterm birth or offspring being LGA in hypertensive mothers, which varied between hypertension types. The exacerbated risks highlight consideration of PCOS in pregnancy counseling and management for women with hypertensive disorders.

**STUDY FUNDING/COMPETING INTEREST(S):**

This study was supported by Shandong Provincial Natural Science Foundation, China [ZR2020MH064 to X.C.], the joint research funding of Shandong University and Karolinska Institute [SDU-KI-2019-08 to X.C. and C.L.], the Finnish Institute for Health and Welfare: Drug and pregnancy project [M.G.], the Swedish Research Council [2022-01188 to C.L.], the regional agreement on medical training and clinical research (ALF) between Stockholm County Council and Karolinska Institute Stockholm County Council [RS2021-0855 to C.L.], the Swedish Brain Foundation [FO2021-0412 to C.L.]. The funders had no role in study design, data collection, analysis, and interpretation, writing of the report or decision to submit for publication. The authors report no conflicts of interest.

**TRIAL REGISTRATION NUMBER:**

N/A.

WHAT DOES THIS MEAN FOR PATIENTS? Women with polycystic ovary syndrome (PCOS) are more likely to develop high blood pressure during pregnancy (gestational hypertension), pre-eclampsia, and chronic hypertension. Although both hypertensive disorders and PCOS are associated with adverse birth outcomes in offspring, it is unknown whether there are synergistic effects (i.e. the overall effect is greater than the sum of each on their own). Based on a nationwide birth cohort from 2004 to 2014 in Finland, this study examined the associations of PCOS with preterm birth and offspring abnormal birth size in hypertensive mothers (mothers with high blood pressure, diagnosed as 140/90 mmHg). The researchers found that PCOS was associated with higher risks of preterm birth in mothers with chronic hypertension and in singleton pregnancies of mothers with pre-eclampsia, while in mothers with gestational hypertension, PCOS was associated with higher risks of offspring born large for gestational age. The researchers concluded that PCOS was associated with increased risks of adverse birth outcomes, which varied between hypertension types. It is crucial for affected women and their physicians to be aware of PCOS-associated additional risks according to specific hypertensive disorders so as to improve birth outcomes through multidisciplinary management.

## Introduction

Hypertensive disorders in pregnancy encompassing chronic hypertension, gestational hypertension, and pre-eclampsia/eclampsia (*de novo* or superimposed on chronic hypertension) (Garovic *et al.*, 2021) are one of the leading pregnancy complications, affecting 7.5–15% of gestations worldwide. Besides effects on maternal health, hypertensive disorders in pregnancy are a major cause of short- and long-term morbidity in the offspring (Garovic *et al.*, 2021). Elevated blood pressure during pregnancy, even below the diagnostic threshold of 140/90 mmHg, is associated with increased preterm birth, infants being small for gestational age (SGA) and low in birthweight (Reddy *et al.*, 2020; Greenberg *et al.*, 2021).

Polycystic ovary syndrome (PCOS) is the most common gynecological endocrine disorder in women of reproductive age, with a prevalence of 5–18% according to the Rotterdam criteria (Joham *et al.*, 2022). The criteria require any two of the following three features for a PCOS diagnosis: clinical or biochemical hyperandrogenism, oligo-ovulation or anovulation, and polycystic ovaries on ultrasound. Hypertensive disorders are overrepresented among women with PCOS. In a non-pregnant setting, the risk for hypertension development increases in women with PCOS from an early age at normal weight and is exacerbated by overweight or obesity (Ollila *et al.*, 2019; Amiri *et al.*, 2020; Mills *et al.*, 2020b; Joham *et al.*, 2021). Among pregnant women with PCOS, a 3- to 4-fold increased risk of gestational hypertension and pre-eclampsia has been reported compared to the non-PCOS counterparts (Bahri Khomami *et al.*, 2019b). Of note, it was estimated that around one-third of women with PCOS had hypertensive disorders according to the traditional threshold of 140/90 mmHg, which reached up to two-thirds if defined by the latest American College of Cardiology/American Heart Association criteria of 130/80 mmHg (Marchesan and Spritzer, 2019; Joham *et al.*, 2021). While hypertensive disorders and PCOS have been revealed to independently increase adverse birth outcomes (Garovic *et al.*, 2021; Farland *et al.*, 2022; Subramanian *et al.*, 2022), it is unknown if comorbid PCOS would exacerbate the risks in hypertensive disorders. Moreover, it is unclear whether PCOS-associated adverse birth outcomes differ between hypertension types.

Taking advantage of a large, nationwide birth cohort in Finland, this study aimed to examine the joint associations of maternal hypertension and PCOS with offspring preterm birth, and being SGA or large for gestational age (LGA).

## Materials and methods

### Ethical consideration

Approvals to use the health data in this study were received from the Finnish authorities providing the data and the data protection authority (the Finnish Institute for Health and Welfare (THL), THL/1662/5.05.00/2015, THL/1853/5.05.00/2016, THL/1496/5.05.00/2019 and addendum), and the Swedish ethics review authority (2023-03041). Because the data were de-identified, informed consent from the children and their mothers was not required according to the Finnish regulations. Data were analyzed between 1 February 2021 and 30 November 2023.

### Study population and data source

All live births in Finland between 1 January 2004 and 31 December 2014 were identified from the Finnish Medical Birth Registry (MBR). The MBR includes information on all live births and stillbirths with a gestational age of ≥22 weeks or a birthweight of ≥500 g since 1987. The coverage of MBR is 100% and most of its variables are highly reliable (Artama *et al.*, 2011). Information on maternal diagnosis was identified by the Finnish Care Registers for Health Care (HILMO) using the *International Statistical Classification of Diseases and Related Health Problems (ICD)*. The HILMO contains data on all in-patient treatments since 1969 and specialized outpatient care since 1998 (Sund, 2012). The ninth revision (ICD-9) was used before the year 1996, and the tenth revision (ICD-10) was used thereafter. Through the personal identification number issued to each citizen and permanent resident, data from different datasets and registries were retrieved and merged. To ensure PCOS diagnosis specificity, mothers were excluded from the study if they had the following diagnoses: pituitary adenoma (ICD-9: 227.3; ICD-10: D35.2), disorders of the pituitary glands including hypo/hyperfunction (ICD-9: 253; ICD-10: E22, E23), disorders of the adrenal glands including congenital adrenal hyperplasia and Cushing’s syndrome (ICD-9: 255; ICD-10: E24/E25/E27), galactorrhea (ICD-9: 611.6; ICD-10: N64.3), suprarenal tumor (ICD-9: 194; ICD-10: C74), and Turner’s syndrome (ICD-9: 758.6; ICD-10: Q96) (Supplementary Table S1).

### Ascertainment of maternal PCOS and hypertensive disorders

PCOS was identified based on a diagnosis of PCOS (ICD-9: 256.4; ICD-10 E28.2) or female infertility associated with anovulation (ICD-9: 628.0; ICD-10 N97.0), given that PCOS is the most common cause for anovulatory infertility. Such a diagnosis was defined as exposure irrespective of year-at-onset because the hormonal and metabolic disturbances of PCOS affect women from adolescence across their life span.

Hypertensive disorders in pregnancy include chronic hypertension, gestational hypertension, and pre-eclampsia (Garovic *et al.*, 2021). Chronic hypertension is blood pressure (BP) ≥140/90 mmHg predating pregnancy or diagnosed before 20 weeks’ gestation, identified based on ICD-10: I10–I13, and O10. Gestational hypertension is BP ≥140/90 mmHg arising *de novo* after 20 weeks of gestation without proteinuria and biochemical or hematological abnormalities, identified based on ICD-10: O13. Pre-eclampsia is new-onset or chronic hypertension combined with proteinuria at 20 weeks or more of gestation, identified according to ICD-10: O11, and O14 (Supplementary Table S1). If a mother had more than one hypertension diagnosis, the exposure was set in the following order: pre-eclampsia, chronic hypertension, and gestational hypertension.

### Birth outcomes

Preterm birth was birth prior to 37 complete gestational weeks, and very preterm birth was birth between 22 and 31 gestational weeks. In analyses of the outcome very preterm birth, births between 32 and 36 gestational weeks were not excluded from the background population. Spontaneous births were births excluding those with planned cesarean section or induced vaginal delivery.

SGA/LGA refers to birthweight/length 2 SDs less/more than the Finnish gestational age- and sex-specific mean (Sankilampi *et al.*, 2013), according to the International Societies of Pediatric Endocrinology and the Growth Hormone Research Society (Clayton *et al.*, 2007).

### Covariates

Maternal and birth characteristics associated with birth outcomes as indicated in previous studies were retrieved from the MBR: offspring birth year, sex, parity, maternal age at birth, maternal country of birth, smoking during pregnancy, marriage at delivery, pre-pregnancy BMI (recorded at first prenatal visit, around 7–10 weeks of gestational age), number of fetuses, use of ART (IVF/ICSI/frozen embryo transfer, check-box in the MBR), and maternal diabetes (ICD-10 codes: E11, E14, O24.1, and O24.4, and/or by purchases of insulin, or oral antidiabetic drugs ATC code: A10B before pregnancy).

### Statistical analysis

First, all included offspring were classified for maternal hypertensive disorders into four groups: no hypertension; gestational hypertension; chronic hypertension before pregnancy; and pre-eclampsia. Then, each group was further classified as two categories according to maternal PCOS (yes/no). Characteristics of the children and their mothers were reported for each group. Interactions between maternal PCOS (yes/no) and hypertension (classified as chronic hypertension, pre-eclampsia, gestational hypertension and no hypertension) were examined for each of the four outcome measures (preterm, very preterm birth, SGA and LGA) by fitting a multiplicative interaction term in Model 1 (see below). For those outcomes with significant interactions, passing the Bonferroni-corrected *P* value-threshold of 0.0125 (0.05 divided by four outcomes), similar analyses but stratified for each hypertension type were performed. Mothers with no hypertension and no PCOS were used as reference, and odds ratios (ORs) and 95% CIs were calculated for preterm and very preterm birth by generalized estimating equation models, and for offspring SGA/LGA by logistic regression. The Bonferroni-corrected *P* value-threshold for the hypertension-stratified analyses was set at 0.0016 (2 × 4 exposures × 4 outcomes; 0.05/32 = 0.0016). The effects of comorbid obesity or diabetes, alongside with the use of ART and multifetal gestations (Bahri Khomami *et al.*, 2019a; Wu *et al.*, 2020; Joham *et al.*, 2021) in the associations, were accounted for by differently adjusted models and subgroup analyses. Specifically, in Model 1, the analyses were adjusted for year of birth (continuous), parity (0 or ≥1), maternal age at birth (continuous), maternal birth country (Finland or not), smoking during pregnancy (yes/no), marital status at delivery (yes/no), and pre-pregnancy BMI (continuous). For preterm birth and infants LGA, diabetes was further adjusted for in Model 2, but not for offspring SGA, given that diabetes was not associated with offspring SGA (Chen *et al.*, 2022). Stratified analyses were then performed by excluding pregnancies conceived with ART (Model 3) or multifetal gestations (Model 4).

To examine spontaneous preterm birth, planned cesarean sections and induced vaginal labor were excluded in a second-round of hypertension-stratified analysis. Finally, sensitivity analyses were conducted after excluding mothers with female infertility associated with anovulation (ICD-9: 628.0; ICD-10: N97.0), to test the robustness of PCOS diagnosis.

Based on the prevalence of preterm (6.7% versus 4.8%) and very preterm birth (0.13% versus 0.06%) in women with and without PCOS, respectively, in previous studies (Valgeirsdottir *et al.*, 2021) and the sample sizes of our study population (17 313 for PCOS, 635 419 for non-PCOS), this study achieved a power of 100% to detect a significant main effect with a two-sided type 1 error of 1%. The power for hypertension-stratified analyses was lower, e.g. 58% to detect a significant difference of preterm birth risk between PCOS and non-PCOS in mothers with chronic hypertension. Missing data were rare in this study, below 5.0% for most variables, and only samples with complete information were included in corresponding analyses. All analyses were performed using SAS, version 9.4 (SAS Institute, Inc, Cary, NC, USA).

## Results

### Demographic characteristics

Of 652 732 children, a total of 43 902 (6.7%) were born to mothers with a hypertensive disorder. Of these, 1709 (3.9%) had mothers comorbid with PCOS. Mothers with a hypertensive disorder were more likely to have multifetal pregnancies, have increased BMI before pregnancy, and receive ART treatment, while they were less likely to experience vaginal delivery compared with mothers without a hypertensive disorder (Table 1).

**Table 1.** Demographic characteristics of all children and their mothers in Finland 2004–2014.

	No hypertension		Chronic hypertension		Gestational hypertension		Pre-eclampsia	
	No PCOS	PCOS	No PCOS	PCOS	No PCOS	PCOS	No PCOS	PCOS
	(n = 593 226)	(n = 15 604)	(n = 10 535)	(n = 532)	(n = 13 668)	(n = 505)	(n = 17 990)	(n = 672)
**Offspring birth year**								
2004–2009	323 172 (54.5)	7286 (46.7)	6768 (64.2)	291 (54.7)	7863 (57.5)	262 (51.9)	10 055 (55.9)	327 (48.7)
2010–2014	270 054 (45.5)	8318 (53.3)	3767 (35.8)	241 (45.3)	5805 (42.5)	243 (48.1)	7935 (44.1)	345 (51.3)
**Offspring sex**								
Boy	303 626 (51.2)	7993 (51.2)	5399 (51.2)	285 (53.6)	7281 (53.3)	290 (57.4)	9091 (50.5)	355 (52.8)
Girl	289 600 (48.8)	7611 (48.8)	5136 (48.8)	247 (46.4)	6387 (46.7)	215 (42.6)	8899 (49.5)	317 (47.2)
**Number of fetuses**								
1	578 465 (97.5)	15 040 (96.4)	10 163 (96.5)	507 (95.3)	13 006 (95.2)	452 (89.5)	15 881 (88.3)	581 (86.5)
≥2	14 761 (2.5)	564 (3.6)	372 (3.5)	25 (4.7)	662 (4.8)	53 (10.5)	2109 (11.7)	91 (13.5)
**Mode of delivery**								
Vaginal	451 655 (76.1)	10 848 (69.5)	6398 (60.7)	281 (52.8)	9147 (66.9)	277 (54.9)	9268 (51.5)	308 (45.8)
Instrumental	48 933 (8.2)	1464 (9.4)	752 (7.1)	40 (7.5)	1509 (11.0)	48 (9.5)	1765 (9.8)	73 (10.9)
Planned CS	38 958 (6.6)	1360 (8.7)	1199 (11.4)	72 (13.5)	907 (6.6)	64 (12.7)	1553 (8.6)	67 (10.0)
Other CS	53 281 (9.0)	1931 (12.4)	2180 (20.7)	139 (26.1)	2104 (15.4)	116 (23.0)	5389 (30.0)	224 (33.3)
Missing	399 (0.1)	1 (0.0)	6 (0.1)	0 (0.0)	1 (0.0)	0 (0.0)	15 (0.1)	0 (0.0)
**Maternal age at delivery, years**								
<25	107 937 (18.2)	1501 (9.6)	790 (7.5)	23 (4.3)	2745 (20.1)	37 (7.3)	3988 (22.2)	74 (11.0)
25–29	188 924 (31.8)	4728 (30.3)	2334 (22.2)	113 (21.2)	4198 (30.7)	146 (28.9)	5607 (31.2)	211 (31.4)
30–34	187 110 (31.5)	5866 (37.6)	3313 (31.4)	181 (34.0)	3869 (28.3)	200 (39.6)	5008 (27.8)	231 (34.4)
≥35	109 255 (18.4)	3509 (22.5)	4098 (38.9)	215 (40.4)	2856 (20.9)	122 (24.2)	3387 (18.8)	156 (23.2)
**Maternal BMI, kg/m^2^**								
<18.5	81 675 (13.8)	1747 (11.2)	420 (4.0)	8 (1.5)	976 (7.1)	21 (4.2)	1678 (9.3)	48 (7.1)
19-24	299 511 (50.5)	6894 (44.2)	2900 (27.5)	81 (15.2)	5549 (40.6)	137 (27.1)	7698 (42.8)	220 (32.7)
25-29	120 376 (20.3)	3526 (22.6)	2700 (25.6)	124 (23.3)	3731 (27.3)	112 (22.2)	4335 (24.1)	151 (22.5)
30-34	41 911 (7.1)	1790 (11.5)	1967 (18.7)	163 (30.6)	1984 (14.5)	120 (23.8)	1997 (11.1)	135 (20.1)
≥35	18 448 (3.1)	995 (6.4)	1879 (17.8)	137 (25.8)	1219 (8.9)	104 (20.6)	1168 (6.5)	77 (11.5)
Missing	31 305 (5.3)	652 (4.2)	669 (6.4)	19 (3.6)	209 (1.5)	11 (2.2)	1114 (6.2)	41 (6.1)
**Parity**								
0	241 090 (40.6)	7068 (45.3)	4379 (41.6)	270 (50.8)	7804 (57.1)	289 (57.2)	11 362 (63.2)	429 (63.8)
≥1	351 692 (59.3)	8531 (54.7)	6146 (58.3)	262 (49.2)	5862 (42.9)	216 (42.8)	6608 (36.7)	243 (36.2)
Missing	444 (0.1)	5 (0.0)	10 (0.1)	0 (0.0)	2 (0.0)	0 (0.0)	20 (0.1)	0 (0.0)
**Maternal country of birth**								
Finnish	535 581 (90.3)	14 257 (91.4)	10 160 (96.4)	507 (95.3)	13 015 (95.2)	480 (95.0)	16 718 (92.9)	628 (93.5)
Non-Finnish	56 036 (9.4)	1346 (8.6)	367 (3.5)	25 (4.7)	639 (4.7)	25 (5.0)	1216 (6.8)	43 (6.4)
Missing	1609 (0.3)	1 (0.0)	8 (0.1)	0 (0.0)	14 (0.1)	0 (0.0)	56 (0.3)	1 (0.1)
**Marital status**								
Married	346 229 (58.4)	10 823 (69.4)	6193 (58.8)	366 (68.8)	7490 (54.8)	333 (65.9)	9518 (52.9)	447 (66.5)
Cohabiting	191 462 (32.3)	3863 (24.8)	3504 (33.3)	133 (25.0)	5101 (37.3)	135 (26.7)	6482 (36.0)	188 (28.0)
Unmarried	53 767 (9.1)	897 (5.7)	812 (7.7)	29 (5.5)	1052 (7.7)	36 (7.1)	1930 (10.7)	36 (5.4)
Missing	1768 (0.3)	21 (0.1)	26 (0.2)	4 (0.8)	25 (0.2)	1 (0.2)	60 (0.3)	1 (0.1)
**Smoking during pregnancy**								
Yes	91 029 (15.3)	1771 (11.3)	1509 (14.3)	56 (10.5)	2258 (16.5)	60 (11.9)	2622 (14.6)	92 (13.7)
No	487 909 (82.2)	13 479 (86.4)	8750 (83.1)	456 (85.7)	11 192 (81.9)	434 (85.9)	14 955 (83.1)	564 (83.9)
Missing	14 288 (2.4)	354 (2.3)	276 (2.6)	20 (3.8)	218 (1.6)	11 (2.2)	413 (2.3)	16 (2.4)
**Diabetes**								
Pre-gestational	5517 (0.9)	401 (2.6)	704 (6.7)	71 (13.3)	244 (1.8)	27 (5.3)	673 (3.7)	41 (6.1)
Gestational	85 295 (14.4)	3224 (20.7)	3226 (30.6)	201 (37.8)	2943 (21.5)	150 (29.7)	3353 (18.6)	203 (30.2)
No	502 414 (84.7)	11 979 (76.8)	6605 (62.7)	260 (48.9)	10 481 (76.7)	328 (65.0)	13 964 (77.6)	428 (63.7)
**ART treatment**								
Yes	11 767 (2.0)	2223 (14.2)	313 (3.0)	81 (15.2)	409 (3.0)	91 (18.0)	880 (4.9)	143 (21.3)
No	581 459 (98.0)	13 381 (85.8)	10 222 (97.0)	451 (84.8)	13 259 (97.0)	414 (82.0)	17 110 (95.1)	529 (78.7)

Data presented as n (%). CS: cesarean section.

### Preterm birth

**Figure 1.**
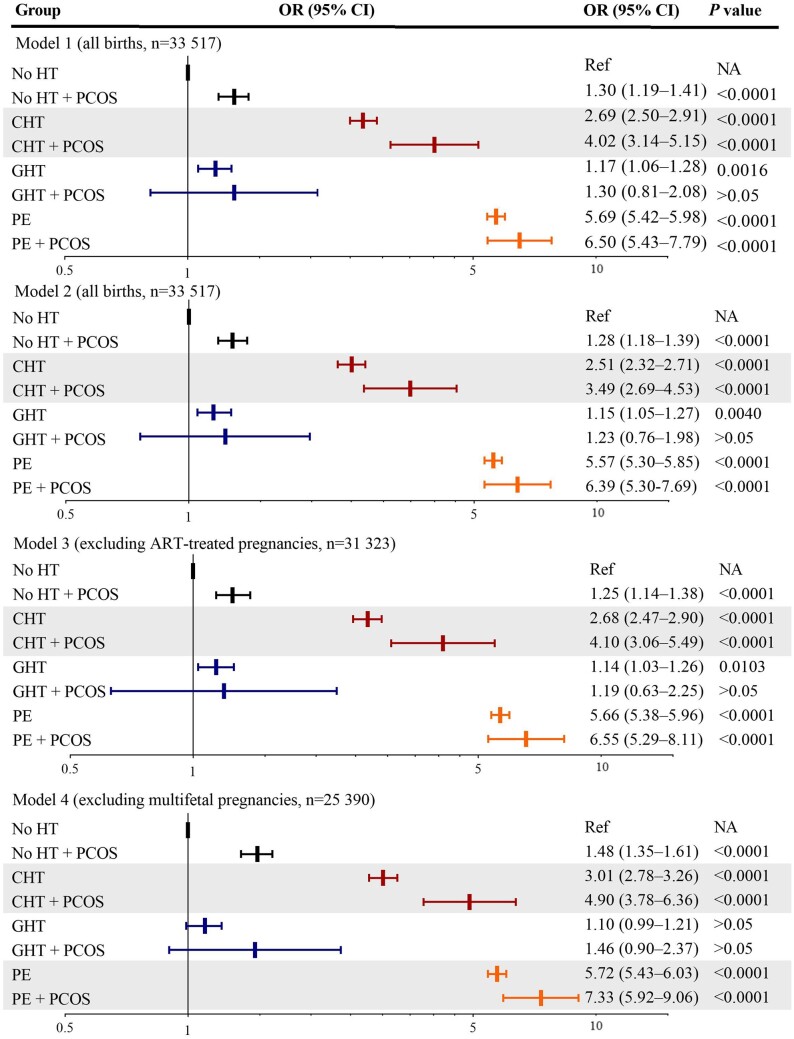
**Synergistic effect modifications of PCOS on the association between chronic hypertension and preterm birth and on the association between pre-eclampsia and preterm birth in singleton pregnancies.** Model 1: analysis in all children, adjusting for birth year, parity, maternal age, country of birth, marital status, smoking, and pre-pregnancy BMI, revealing an interaction between PCOS and chronic hypertension on preterm birth (*P*_interaction_ < 0.001). Model 2: analysis in all children, adjusting for covariates in Model 1, plus pre- and gestational diabetes. Model 3: excluding ART-conceived pregnancies, adjusting for covariates in Model 1. Model 4: excluding multifetal gestations, adjusting for covariates in Model 1, detecting an effect modification for PCOS on the association between pre-eclampsia and preterm birth (*P*_interaction_ < 0.001). OR: odds ratio; HT, hypertensive disorder; CHT, chronic hypertension; PE, pre-eclampsia; GHT, gestational hypertension; Ref, reference; NA, not available. Preterm births are all births before 37 gestational weeks.

Among mothers with chronic hypertension, pre-eclampsia, and gestational hypertension, the proportions of offspring prematurity were higher for those with PCOS than those without (Supplementary Table S2). Statistically significant interactions were observed for PCOS (yes/no) with hypertension (chronic hypertension/pre-eclampsia/gestational hypertension/no) on preterm birth (*F *=* *504.1, *P*_interaction_ < 0.001). Therefore, hypertension-stratified analyses were performed. Using no hypertension and no PCOS as reference and adjusting for birth year, parity, maternal age, country of birth, marital status, smoking, and pre-pregnancy BMI (Model 1), the risk of preterm birth was overrepresented in mothers with pre-eclampsia and chronic hypertension. Maternal PCOS alone was associated with 30% increased risks of preterm birth (adjusted odds ratio (aOR) 1.30, 95% CI 1.19–1.41). Notably, in chronic hypertension, the risk of preterm birth was significantly higher when comorbid with PCOS (aOR_PCOS_ 4.02, 3.14–5.15 versus aOR_non-PCOS_ 2.69, 2.50–2.91), which remained higher after diabetes was further adjusted for (Model 2), excluding ART-conceived (Model 3) or excluding multifetal gestations (Model 4). A statistically significant interaction was observed between chronic hypertension and PCOS on preterm birth (*F *=* *32.7, *P*_interaction_ < 0.001). Also, a higher risk of preterm birth was revealed in singleton pregnancies (Model 4) for PCOS (aOR 7.33, 5.92–9.06) than non-PCOS (aOR 5.72, 5.43–6.03) among mothers with pre-eclampsia (Fig. 1, Supplementary Table S2), and a significant effect modification was detected for PCOS on the association between pre-eclampsia and preterm birth (*F *=* *50.0, *P*_interaction_ < 0.001).

### Very preterm birth

**Figure 2.**
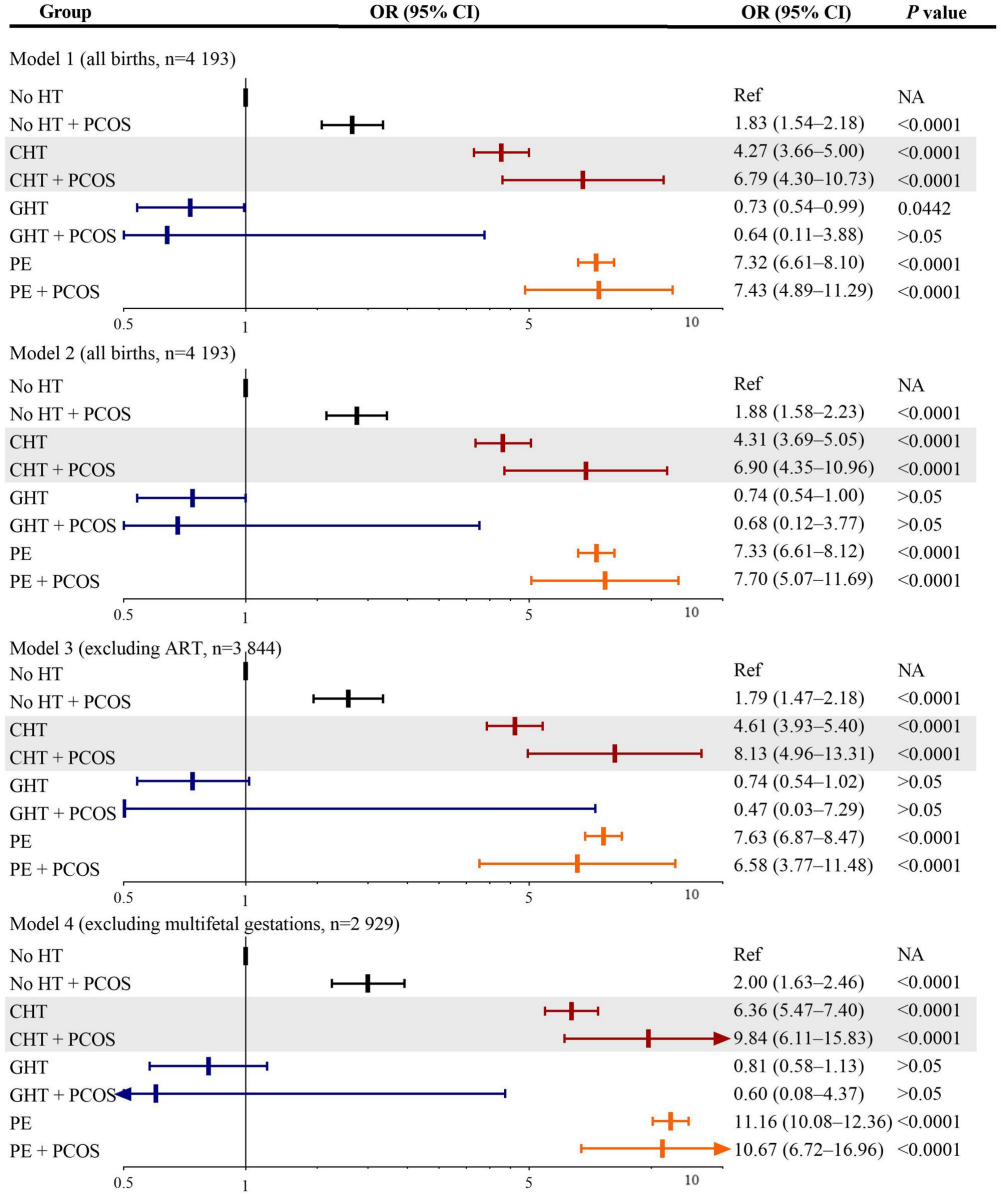
**Association for PCOS and chronic hypertension with very preterm birth.** Model 1: analysis in all children, adjusting for birth year, parity, maternal age, country of birth, marital status, smoking, and pre-pregnancy BMI. Model 2: analysis in all children, adjusting for covariates in Model 1, plus pre- and gestational diabetes. Model 3: excluding ART-conceived pregnancies, adjusting for covariates in Model 1. Model 4: excluding multifetal gestations, adjusting for covariates in Model 1. OR: odds ratio; HT, hypertensive disorder; GHT, gestational hypertension; CHT, chronic hypertension; PE, pre-eclampsia; Ref, reference; NA, not available. Very preterm births are all births before 32 gestational weeks.

In addition, there were significant interactions on very preterm birth between PCOS and hypertension (*F *=* *124.2, *P*_interaction_ < 0.001). Analyses stratified by hypertension type revealed that the risk of very preterm birth tended to be higher for those with PCOS than those without PCOS among mothers with chronic hypertension (Models 1–3). Among mothers with pre-eclampsia or gestational hypertension, there was no additional risks of very preterm birth associated with PCOS (Fig. 2, Supplementary Table S2).

### Offspring birth size

**Figure 3.**
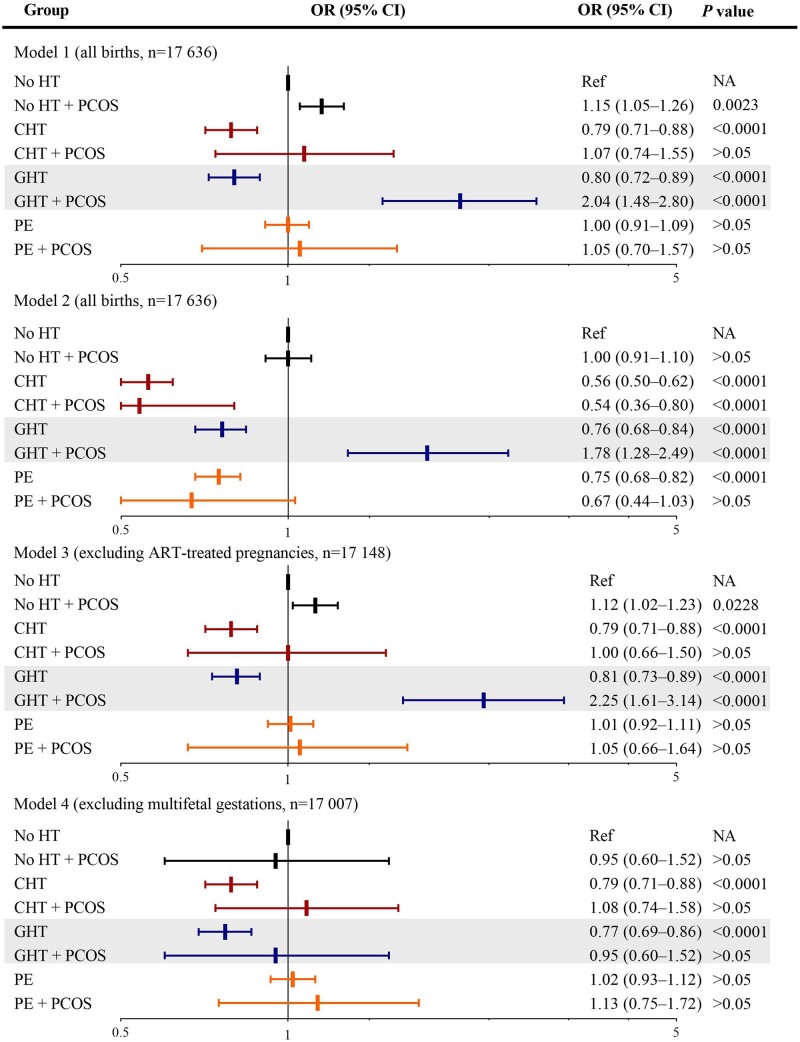
**Synergistic effect modification of PCOS on the association between gestational hypertension and offspring large for gestational age.** Model 1: analysis in all children, adjusting for birth year, parity, maternal age, country of birth, marital status, smoking, and pre-pregnancy BMI, revealing an effect modification for PCOS on the association between gestational hypertension and LGA (*P*_interaction_ = 0.002). Model 2: analysis in all children, adjusting for covariates in Model 1, plus pre- and gestational diabetes. Model 3: excluding ART conceived pregnancies, adjusting for covariates in Model 1. Model 4: excluding multifetal gestations, adjusting for covariates in Model 1. OR: odds ratio; HT, hypertensive disorder; GHT, gestational hypertension; CHT, chronic hypertension; PE, pre-eclampsia; Ref, reference; NA, not available. Large for gestational age refers to birthweight/length 2 SDs more than the Finnish gestational age- and sex-specific mean (Sankilampi *et al.*, 2013), according to the International Societies of Pediatric Endocrinology and the Growth Hormone Research Society (Clayton *et al.*, 2007).

Significant interactions were observed on offspring SGA between PCOS and hypertension (*F *=* *99.5, *P*_interaction_ < 0.001). Stratified analysis showed that although there were overrepresented risks of offspring SGA across all hypertensive mothers compared with mothers with no hypertensive disorder and no PCOS, the effect sizes were comparable between those with and without PCOS within each type of hypertension (Supplementary Table S3). For offspring LGA, there were also significant interactions between PCOS and hypertension (*F *=* *2.7, *P*_interaction_ = 0.012). Stratified analyses revealed that in mothers with gestational hypertension, there was a 2-fold higher risk for offspring LGA in those with PCOS, which was significantly higher than that without PCOS (Models 1–3), with a significant effect modification for PCOS on the association between gestational hypertension and LGA (*F *=* *9.7, *P*_interaction_ = 0.002), whereas in mothers with chronic hypertension or pre-eclampsia, the effect sizes for offspring LGA were comparable between those with and without PCOS (Fig. 3, Supplementary Table S2).

### Sensitivity analyses

**Figure 4.**
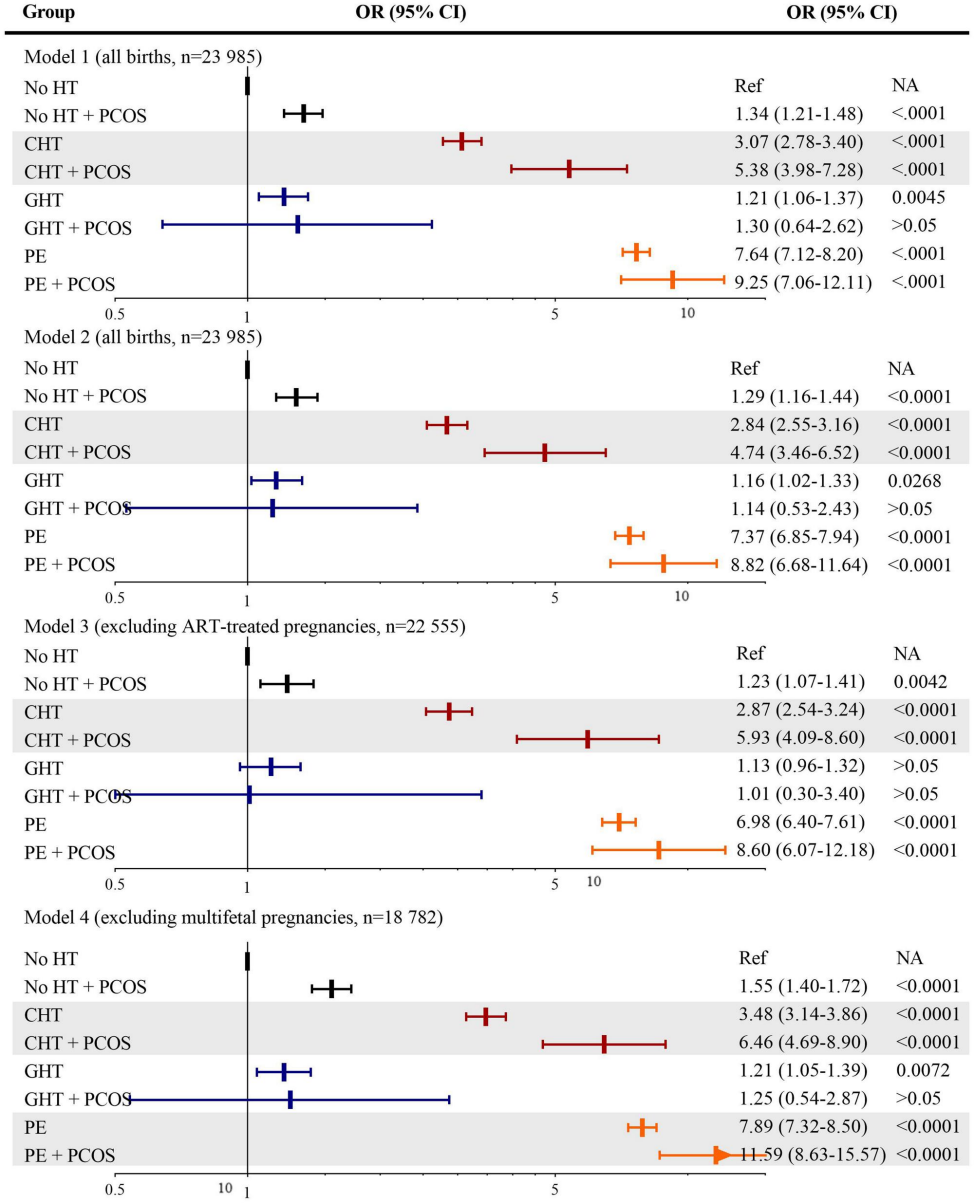
**Synergistic effect modifications of PCOS on the association between chronic hypertension and spontaneous preterm birth and on the association between pre-eclampsia and spontaneous preterm birth in singleton pregnancies.** Model 1: analysis in all children, adjusting for birth year, parity, maternal age, country of birth, marital status, smoking, and pre-pregnancy BMI, detecting an effect modification between PCOS and chronic hypertension on spontaneous preterm birth (*P*_interaction_ < 0.01). Model 2: analysis in all children, adjusting for covariates in Model 1, plus pre- and gestational diabetes. Model 3: excluding ART conceived pregnancies, adjusting for covariates in Model 1. Model 4: excluding multifetal gestations, adjusting for covariates in Model 1, revealing an effect modification between PCOS and pre-eclampsia on spontaneous preterm birth (*P*_interaction_ < 0.001). OR: odds ratio; HT, hypertensive disorder; GHT, gestational hypertension; CHT, chronic hypertension; PE, pre-eclampsia; Ref, reference; NA, not available. Spontaneous preterm births are all births before 37 gestational weeks, excluding planned cesarean section and induced vaginal delivery.

**Figure 5.**
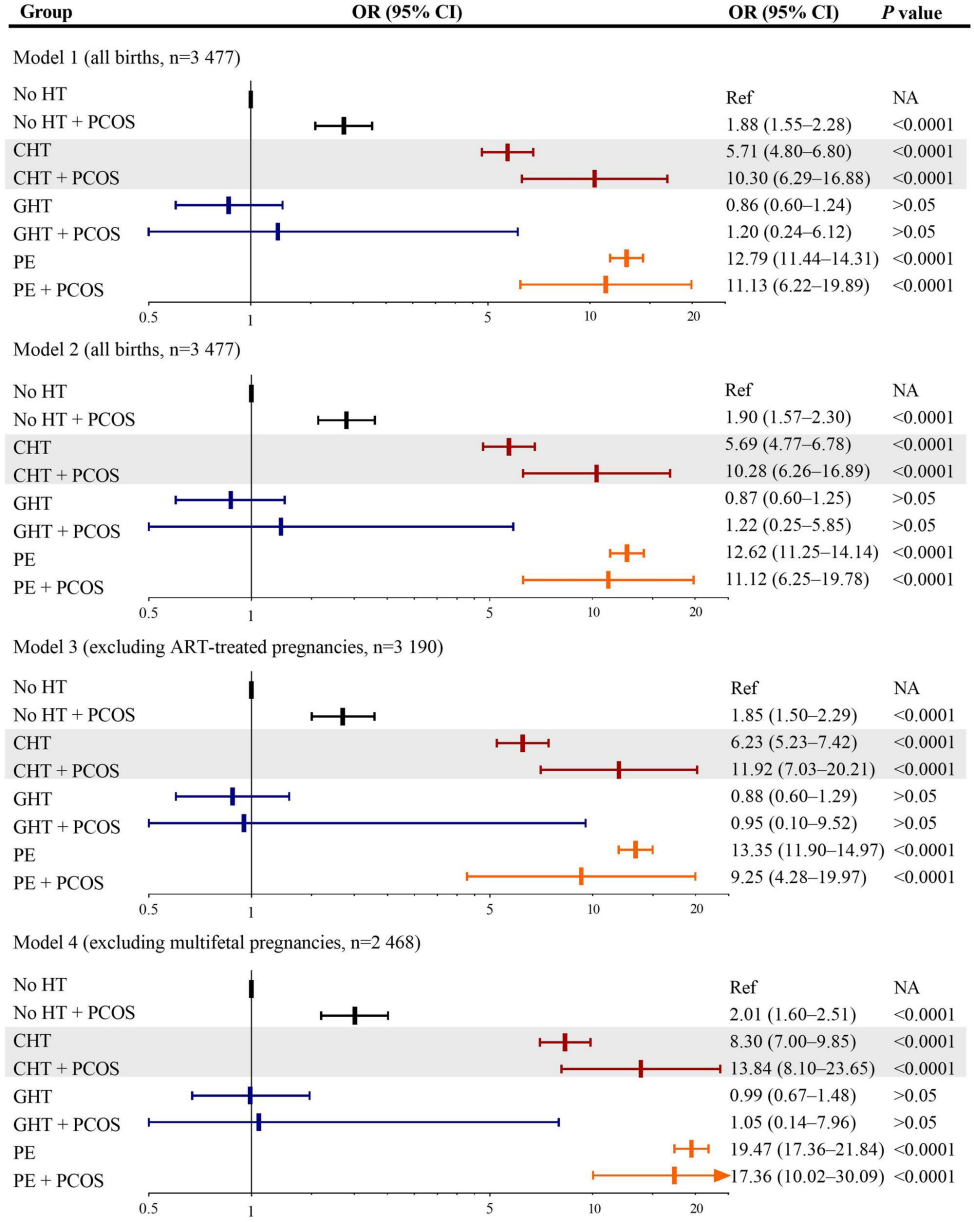
**Synergistic effect modification of PCOS on the association between chronic hypertension and spontaneous very preterm birth.** Model 1. Analysis in all children, adjusting for birth year, parity, maternal age, country of birth, marital status, smoking, and pre-pregnancy BMI, revealing an effect modification between PCOS and chronic hypertension on spontaneous preterm birth (*P*_interaction_ < 0.01). Model 2. Analysis in all children, adjusting for covariates in Model 1, plus pre- and gestational diabetes. Model 3. Excluding ART-conceived pregnancies, adjusting for covariates in Model 1. Model 4. Excluding multifetal gestations, adjusting for covariates in Model 1. OR: odds ratio; HT, hypertension; GHT, gestational hypertension; CHT, chronic hypertension; PE, pre-eclampsia; Ref, reference; NA, not available. Spontaneous very preterm births were all births before 32 gestational weeks, excluding planned cesarean section and induced vaginal delivery.

In subgroup analyses of spontaneous births, significant interactions were detected between PCOS and hypertension on spontaneous preterm (*F *=* *384.0, *P*_interaction_ < 0.001) and very preterm birth (*F *=* *149.8, *P*_interaction_ < 0.001). Hypertension-stratified analyses showed a pattern of associations for spontaneous preterm and very preterm birth similar to that for total preterm birth, yet with larger effect sizes (Figs 4 and 5, Supplementary Table S2).

Excluding mothers with anovulatory infertility confirmed the additional risks associated with PCOS in mothers with chronic hypertension for very and spontaneous very preterm birth, while the combined association of PCOS and gestational hypertension with LGA did not persist (Supplementary Tables S4, S5, and S6).

## Discussion

This population-based cohort study found that PCOS was associated with: higher risks of preterm birth for mothers with chronic hypertension; higher risks of preterm birth in singleton pregnancies of mothers with pre-eclampsia; and higher risks of offspring LGA in mothers with gestational hypertension. Moreover, the associations of PCOS with preterm birth in chronic hypertension or pre-eclampsia, were found also in the spontaneous birth cohort. It is crucial for affected women and their physicians to be aware of the PCOS-associated additional risks according to specific hypertensive disorders so as to improve birth outcomes through multidisciplinary management.

We found that the odds ratios for preterm birth were increased in mothers with chronic hypertension, and significantly more so when comorbid with PCOS. Notably, the PCOS-associated prematurity risks tended to be higher at earlier gestational ages, reaching up to 10-fold for very preterm birth in chronic hypertension with PCOS. Preterm birth is a worldwide problem with not only increased risks of neonatal morbidity and mortality, but also adverse health consequences during childhood and adulthood. An awareness of increased preterm birth in mothers with PCOS and chronic hypertension is therefore essential, particularly given that the incidences of both are still increasing (Joham *et al.*, 2022; Cameron *et al.*, 2023). This was the first study, to the best of our knowledge, to examine preterm birth in relation to maternal PCOS in the setting of chronic hypertension. However, our findings were consistent with recent large cohort studies which revealed an increased risk of preterm birth in mothers with PCOS after adjusting for pre-existing and pregnancy-induced hypertensive disorders among many other covariates (Mills *et al.*, 2020a; Valgeirsdottir *et al.*, 2021). A novel finding in our study was that PCOS and chronic hypertension were synergistically associated with higher risks of preterm birth, yet the underlying mechanisms remain unknown. In the current study, almost 40% of the mothers with chronic hypertension were obese and a quarter were comorbid with diabetes. Meanwhile, metabolic disturbances have been increasingly recognized in PCOS, even in non-obese patients (Zhu *et al.*, 2019). It is possible that metabolic impairments are exacerbated in comorbid PCOS and chronic hypertension, which might be associated with more severe vasculopathy. Poor perfusion and accelerated aging of the placenta would, in turn, initiate an early onset of delivery.

In mothers with pre-eclampsia, more preterm births were also expected for those with PCOS, as both disorders are associated with placental dysfunction and increased androgens (Brosens *et al.*, 2011; Kelley *et al.*, 2019). Our study indeed found a higher risk of preterm birth in mothers affected by both disorders than in those affected by pre-eclampsia alone, including a synergistic interaction between the exposures, but only in singletons. In agreement, a cohort study demonstrated a higher rate of preterm delivery in singleton than multifetal pregnancies of mothers with PCOS (Mills *et al.*, 2020a). Conversely, there was a study reporting more adverse obstetric outcomes for multiple than singleton gestations in women with PCOS (Peeva *et al.*, 2023). Our sample size was not powered for a subgroup analysis in twins of mothers with pre-eclampsia and PCOS. Future studies are warranted to elucidate whether the effect of PCOS on birth outcomes is mechanistically different between singleton and multifetal gestations in the setting of maternal pre-eclampsia.

Importantly, this study demonstrated that in chronic hypertension and pre-eclampsia, the association pattern of PCOS with preterm birth also applied to spontaneous births, with larger effect sizes than that of total preterm birth. Our findings were in line with a study in twin pregnancies of mothers with PCOS, which revealed higher risks of spontaneous than total preterm birth (Løvvik *et al.*, 2015). Also, studies reported that preterm delivery in women with PCOS was predominantly due to preterm labor and preterm premature rupture of membranes (Yamamoto *et al.*, 2012; Valgeirsdottir *et al.*, 2021). Taken together, these results indicated an important role of PCOS in spontaneous preterm birth.

We found 2-fold increased risks for offspring LGA in mothers with gestational hypertension and PCOS, with a synergistic interaction between the exposures, while neither PCOS nor gestational hypertension alone was associated with offspring LGA after considering maternal BMI, and pre-gestational and gestational diabetes. A possible explanation could be that PCOS symptoms are exacerbated by factors contributing to the development of gestational hypertension: for example, maternal obesity and hyperglycemia, which in turn promotes excessive *in utero* growth (HAPO Study Cooperative Research Group, 2009). Mothers with PCOS are at higher risks of impaired glucose tolerance independent of a diabetes diagnosis (Gilbert *et al.*, 2018) and they are more likely to gain excessive weight during pregnancy (Bahri Khomami *et al.*, 2019a). However, we noted that the synergistic effects of PCOS and gestational hypertension on LGA were not seen when mothers with anovulatory infertility were excluded. Further studies are warranted to elucidate if the latter is attributed to PCOS phenotypes, the reduced sample size or other factors.

The main strength of this study lies in the large nationwide population-based registries. The comprehensive datasets with validated records (Sund, 2012) made it possible to perform multiple adjustments and model constructions. We evaluated both total and spontaneous preterm birth stratified by severity. Moreover, SGA and LGA are determined based on both birthweight and length. While prior studies also considered BMI as an important covariate, we additionally accounted for maternal diabetes, fertility treatment, and multifetal gestations.

The limitations of our study should be recognized. First, data on PCOS subtypes, antihypertensive medication use during pregnancy, and onset date of pre-eclampsia or gestational hypertension were not available. Also, we had no information on prescription of metformin or other insulin sensitizers during pregnancy. Maternal BMI was recorded once, and data on weight gain during pregnancy were unavailable. Second, all diagnoses were retrieved from registries based on ICD codes. Our study might predominantly capture those with more severe symptoms who sought medical care, thus overestimating the associations. Meanwhile, women with PCOS not seeking medical care for their symptoms would be misclassified in the control group, resulting in underestimated effect sizes of the associations. Third, inclusion of anovulatory infertility as PCOS might introduce diagnosis bias, although PCOS constituted around 80% of anovulatory infertility (Balen *et al.*, 2016) and our sensitivity analysis produced similar results to the main analysis. Fourth, despite the total number being large in our study, only 505 to 672 pregnancies were with PCOS among hypertensive mothers, which restricted the power to detect differences between groups. This was particularly the case for very preterm birth. Also, multifetal pregnancies among maternal PCOS were too few to be analyzed separately. Fifth, ART included IVF/ICSI only, which was not the first-line treatment for fertility in women with PCOS (Escobar-Morreale, 2018). Potential effects of other treatments, such as ovulation induction, could not be ruled out.

## Conclusion

PCOS was associated with higher risks of preterm birth in mothers with chronic hypertension and in singleton pregnancies of mothers with pre-eclampsia, and increased risks of offspring being LGA in mothers with gestational hypertension. The differential association of maternal PCOS with adverse birth outcomes between hypertension types may have implications for pre-conception counseling and pregnancy management in affected women.

## Supplementary Material

hoad048_Supplementary_Data

## Data Availability

Data are available with the permission of Finnish Social and Health Data Permit Authority (findata.fi).
