# Supplementary material for: Birth outcomes in mothers with hypertensive disorders and polycystic ovary syndrome: a population-based cohort study
Source: Hum Reprod Open. 2023 Dec 4;2023(4):hoad048. doi: 10.1093/hropen/hoad048 (PMC10919338; doi:10.1093/hropen/hoad048)
Supplement: hoad048_Supplementary_Data [file hoad048_supplementary_data.doc]

**Supplementary Table S1** Summary of ICD and ATC codes in the analyses.

| Diagnosis | Codes |
| --- | --- |
| PCOS | ICD-9: 256.4, 628.0, ICD-10: E28.2, N97.0 |
| Chronic hypertension | ICD-10: I10–13, O10 |
| Gestational hypertension | ICD-10: O13 |
| Preeclampsia | ICD-10: O11, and O14 |
| Diabetes | ICD-10: O24.4, ICD-10: E11, E14, and O24.1, and/or by purchases of insulin, or oral antidiabetic drugs ATC A10B before pregnancy. |
| Pituitary adenoma | ICD-9: 227.3; ICD-10: D35.2 |
| Pituitary hypo/hyperfunction | ICD-9: 253; ICD-10: E22, E23 |
| Congenital adrenal hyperplasia and Cushing’s syndrome | ICD-9: 255; ICD-10: E24/E25/E27 |
| Galactorrhea | ICD-9: 611.6; ICD-10: N64.3 |
| Suprarenal tumor | ICD-9: 194; ICD-10: C74 |
| Turner’s syndrome | ICD-9: 758.6; ICD-10: Q96 |

ATC: Anatomical Therapeutic Chemical

ICD: International Statistical Classification of Diseases and Related Health Problems

**Supplementary Table S2** Gestational age, birthweight, SGA and LGA of infants stratified by maternal hypertensive disorders and PCOS.

|  | No HT | | | Chronic HT | | | Gestational HT | | | Preeclampsia | | |
| --- | --- | --- | --- | --- | --- | --- | --- | --- | --- | --- | --- | --- |
|  | No PCOS (n=593 226) | PCOS (n=15 604) | p value | No PCOS (n=10 535) | PCOS (n=532) | p value | No PCOS (n=13 668) | PCOS (n=505) | p value | No PCOS (n=17 990) | PCOS (n=672) | p value |
| Mean ±SD, weeks | 40.0 ±3.5 | 39.5 ±2.3 | ＜0.001 | 39.1 ±4.2 | 38.5 ±2.7 | 0.001 | 39.6 ±2.8 | 39.2 ±1.9 | 0.001 | 38.2 ±4.2 | 37.9 ±2.9 | >0.05 |
| Preterm birth | 27 688 (4.7) | 1157 (7.4) | ＜0.001 | 1393 (13.2) | 95 (17.9) | 0.002 | 829 (6.1) | 43 (8.5) | 0.024 | 4431 (24.6) | 186 (27.7) | >0.05 |
| Very preterm birth | 3289 (0.6) | 185 (1.2) | ＜0.001 | 274 (2.6) | 21 (3.9) | >0.05 | 60 (0.4) | 3 (0.6) | >0.05 | 781 (4.3) | 31 (4.6) | >0.05 |
| Spontaneous preterm birth | 20 934 (4.6) | 872 (7.9) | ＜0.001 | 850 (15.4) | 59 (21.9) | 0.013 | 437 (6.3) | 23 (10.1) | >0.05 | 2498 (31.9) | 102 (37.5) | >0.05 |
| Spontaneous very preterm birth | 2784 (0.6) | 153 (1.4) | ＜0.001 | 216 (3.9) | 18 (6.7) | 0.037 | 43 (0.6) | 3 (1.3) | >0.05 | 635 (8.1) | 23 (8.5) | >0.05 |
| Mean ±SD, g | 3529 ±570 | 3489 ±604 | ＜0.001 | 3292 ±747 | 3241 ±772 | >0.05 | 3392 ±594 | 3401 ±615 | >0.05 | 3044 ±825 | 3070 ±861 | >0.05 |
| SGA | 16 555 (2.8) | 436 (2.8) | >0.05 | 921 (8.7) | 45 (8.5) | >0.05 | 968 (7.1) | 36 (7.1) | >0.05 | 2171 (12.1) | 61 (9.1) | 0.019 |
| LGA | 15 651 (2.6) | 551 (3.5) | ＜0.001 | 404 (3.8) | 32 (6.0) | 0.012 | 378 (2.8) | 44 (8.7) | ＜0.001 | 550 (3.1) | 26 (3.9) | >0.05 |

Data presented as mean ±SD or n (%). Comparison between groups did not adjust for covariates or include interaction terms. HT, hypertension. Preterm birth: births before 37 gestational weeks. Spontaneous preterm birth was all preterm births excluding planned cesarean section and induced vaginal labor. Small/large for gestational age (SGA/LGA) refers to birthweight/length two standard deviations less/more than the Finnish gestational age- and sex-specific mean, according to the International Societies of Pediatric Endocrinology and the Growth Hormone Research Society.

**Supplementary Table S3** Adjusted odds ratio for offspring SGA in relation to maternal hypertensive disorders and PCOS.

|  | Model 1 (n=21 193) | | Model 2 (n=20 203) | | Model 3 (n=20 554) | |
| --- | --- | --- | --- | --- | --- | --- |
|  | No PCOS | Maternal PCOS | No PCOS | Maternal PCOS | No PCOS | Maternal PCOS |
| No HP | 1.00 | 1.02 (0.93–1.13) | 1.00 | 1.00 (0.91–1.11) | 1.00 | 1.02 (0.92–1.14) |
| Chronic HT | 4.27 (3.96–4.60) ^*^ | 4.25 (3.09–5.85) ^*^ | 4.40 (4.08–4.75) ^*^ | 4.34 (3.13–6.03) ^*^ | 4.31 (4.00–4.65) ^*^ | 4.70 (3.36–6.59) ^*^ |
| Gestational HT | 2.85 (2.66–3.05) ^*^ | 3.32 (2.36–4.68) ^*^ | 2.95 (2.75–3.16) ^*^ | 3.76 (2.66–5.31) ^*^ | 2.86 (2.66–3.06) ^*^ | 3.93 (2.74–5.63) ^*^ |
| Preeclampsia | 4.86 (4.62–5.11) ^*^ | 3.85 (2.94–5.05) ^*^ | 5.30 (5.03–5.58) ^*^ | 4.39 (3.31–5.82) ^*^ | 4.94 (4.70–5.20) ^*^ | 4.40 (3.27–5.91) ^*^ |

Data were presented as OR (95% CI). ^*^ Significant according to the Bonferroni-corrected *P* value. HT, hypertension. Small for gestational age (SGA) refers to birth weight/length 2 standard deviations <the Finnish gestational age- and sex-specific mean, according to the International Societies of Pediatric Endocrinology and the Growth Hormone Research Society. Model 1: analysis in all children, adjusting for offspring birth year, parity, and maternal age, country of birth, marital status, smoking during pregnancy and maternal pre–pregnancy BMI. Model 2: analysis in singletons, adjusting for covariates in model 1. Model 3: analysis in children excluding use of assisted reproductive technology, adjusting for covariates in model 1.

**Supplementary Table S4** Adjusted odds ratios of preterm and very preterm birth in relation to maternal hypertensive disorders and PCOS excluding mothers with anovulatory infertility.

|  | Preterm birth (n=32 469) | | Very preterm birth (n=4031) | |
| --- | --- | --- | --- | --- |
|  | No PCOS | Maternal PCOS | No PCOS | Maternal PCOS |
| No HT | 1.00 | 1.20 (1.02–1.41) | 1.00 | 1.78 (1.27–2.51) ^*^ |
| Chronic HT | 2.69 (2.49–2.91) ^*^ | 3.41 (2.11–5.52) ^*^ | 4.27 (3.66–4.99) ^*^ | 10.35 (4.92–21.76) ^*^ |
| Gestational HT | 1.17 (1.07–1.29) ^*^ | 1.48 (0.61–3.60) | 0.74 (0.54–1.00) | 0.57 (0.01–48.78) |
| Preeclampsia | 5.70 (5.43–5.99) ^*^ | 7.15 (5.10–10.01) ^*^ | 7.35 (6.65–8.14) ^*^ | 4.87 (2.00–11.86) ^*^ |

Data were presented as OR (95% CI). ^*^ Significant according to the Bonferroni-corrected *P* value. HT, hypertension. Preterm birth: births before 37 gestational weeks. Very preterm birth: births before 32 gestational weeks. Spontaneous preterm birth was all preterm births excluding planned cesarean section and induced vaginal labor. Spontaneous very preterm birth: spontaneous preterm birth before 32 gestational weeks. The analysis was adjusted for offspring birth year, parity, and maternal age, maternal country of birth, marital status, smoking during pregnancy and maternal pre–pregnancy BMI.

**Supplementary Table S5** Adjusted odds ratios of spontaneous preterm and very preterm birth in relation to maternal hypertensive disorders and PCOS excluding mothers with anovulatory infertility.

|  | Spontaneous preterm birth (n=23 256) | | Spontaneous very preterm birth (n=3345) | |
| --- | --- | --- | --- | --- |
|  | No PCOS | Maternal PCOS | No PCOS | Maternal PCOS |
| No HT | 1.00 | 1.39 (1.15–1.69) ^*^ | 1.00 | 1.95 (1.36–2.79) ^*^ |
| Chronic HT | 3.00 (2.70–3.34) ^*^ | 5.32 (2.85–9.92) ^*^ | 5.73 (4.82–6.82) ^*^ | 16.42 (7.94–33.95) ^*^ |
| Gestational HT | 1.19 (1.04–1.36) | 2.04 (0.69–6.00) | 0.87 (0.61–1.26) | 1.63 (0.13–19.71) |
| Preeclampsia | 7.45 (6.91–8.03) ^*^ | 11.00 (6.46–18.72) ^*^ | 12.87 (11.51–14.39) ^*^ | 5.37 (1.46–19.76) ^*^ |

Data were presented as OR (95% CI). ^*^ Significant according to the Bonferroni-corrected *P* value.. HT, hypertension. Preterm birth: births before 37 gestational weeks. Very preterm birth: births before 32 gestational weeks. Spontaneous preterm birth was all preterm births excluding planned cesarean section and induced vaginal labor. Spontaneous very preterm birth: spontaneous preterm birth before 32 gestational weeks. The analysis was adjusted for offspring birth year, parity, and maternal age, maternal country of birth, marital status, smoking during pregnancy and maternal pre–pregnancy BMI.

**Supplementary Table S6** Offspring SGA and LGA in relation to maternal PCOS and hypertensive disorders of pregnancy, excluding mothers with anovulatory infertility.

|  | SGA (n=20 785) | | LGA (n=17 172) |
| --- | --- | --- | --- |
|  | No PCOS | Maternal PCOS | No PCOS Maternal PCOS |
| No HT | 1.00 | 0.99 (0.82–1.20) | 1.00 1.18 (1.00–1.38) |
| Chronic HT | 4.61 (3.97–4.61) ^*^ | 5.88 (3.48–9.93) ^*^ | 0.79 (0.71–0.88) ^*^ 0.60 (0.26–1.37) |
| Gestational HT | 2.85 (2.66–3.05) ^*^ | 2.74 (1.39–5.40) ^*^ | 0.80 (0.72–0.89) ^*^ 1.12 (0.54–2.32) |
| Preeclampsia | 4.87 (4.63–5.12) ^*^ | 4.49 (2.74–7.36) ^*^ | 1.00 (0.92–1.10) 1.49 (0.80–2.78) |

Data were presented as OR (95% CI). ^*^ Significant according to the Bonferroni-corrected *P* value. HT, hypertension. Small/large for gestational age (SGA/LGA) refers to birth weight/length 2 standard deviations less than the Finnish gestational age- and sex-specific mean, according to the International Societies of Pediatric Endocrinology and the Growth Hormone Research Society. The analysis was adjusted for offspring birth year, parity, and maternal age, maternal country of birth, marital status, smoking during pregnancy and maternal pre–pregnancy BMI.
